# Melatonin attenuates palmitic acid-induced mouse granulosa cells apoptosis via endoplasmic reticulum stress

**DOI:** 10.1186/s13048-019-0519-z

**Published:** 2019-05-10

**Authors:** Zhi Chen, Lanjie Lei, Di Wen, Lei Yang

**Affiliations:** 1 College of Biological Science and Agriculture, Qiannan Normal University for Nationalities, Guizhou, 558000 Duyun China; 2 Affiliated Hospital of Jiujiang University, Jiujiang University, Jiujiang, 332000 Jiangxi China; 3 Key Laboratory of System Bio-medicine of Jiangxi Province, Jiujiang University, Jiujiang, 332000 Jiangxi China; 4 College of Basic Medical Science, Jiujiang University, Jiujiang, 332000 Jiangxi China

**Keywords:** Palmitic acid, Melatonin, Endoplasmic reticulum stress, Mouse granulosa cell, Apoptosis

## Abstract

**Background:**

Palmitic acid (PA), the main component of dietary saturated fat, causes apoptosis in many cell types, including mouse granulosa cell. Melatonin, an important endogenous hormone, has beneficial effects on female reproductive processes. Since elevated PA levels are present in follicular fluid (FF) of patients with infertility and are shown to be toxic for granulosa cells, we investigated the molecular mechanisms of PA toxicity in mouse granulosa cells and explored the effects of melatonin on PA-induced apoptosis.

**Methods:**

Granulosa cells from immature female mice were cultured for 24 h in medium containing PA and/or melatonin. Then, the effects of PA alone or combined with melatonin on viability, apoptosis and endoplasmic reticulum (ER) stress in granulosa cells were detected by methyl thiazolyl tetrazolium (MTT) assay, flow cytometry assay and western blot. After 48 h of PA and/or melatonin treatment, the concentrations of estradiol (E2) and progesterone (P4) in the culture supernatants were measured with ELISA kits.

**Results:**

In this study, we explored the effects of melatonin on cell viability and apoptosis in PA-treated mouse granulosa cells and uncovered the signaling pathways involved in these processes. Our results showed that 200-800 μM PA treatment reduces cell viability, induces cell apoptosis, enhances the expression of apoptosis-related genes (Caspase 3 and B-cell lymphoma-2 (BCL-2) associated X protein (BAX)), and activates the expression of ER stress marker genes (glucose-regulated protein 78 (GRP78) and CCAAT/enhancer binding protein homologous protein (CHOP)). Melatonin treatment (1-10 μM) suppresses 400 μM PA-induced cell viability decrease, cell apoptosis, Caspase 3 activation, and BAX, CHOP, and GRP78 expression. In addition, we found that 10 μM melatonin successfully attenuated the 400 μM PA-induced estrogen (E2) and progesterone (P4) decreases.

**Conclusions:**

This study suggests that PA triggers cell apoptosis via ER stress and that melatonin protects cells against apoptosis by inhibiting ER stress in mouse granulosa cells.

**Electronic supplementary material:**

The online version of this article (10.1186/s13048-019-0519-z) contains supplementary material, which is available to authorized users.

## Background

Palmitic acid (PA) is one of the most common fatty acids in animal and human follicular fluid (FF) and blood serum [1–3]. The PA level in mammalian FF is reported to be approximately 10^− 4^ M [3–5]. Recently, increasing evidence has shown that elevated PA levels may be associated with infertility in humans [6, 7]. Animal model studies have reported relations between higher PA levels and decreased rates of fertilization, cleavage, and blastocyst formation [3, 8, 9]. Granulosa cells play essential roles in follicular development, oocyte maturation and sex hormone secretion [10–12]. The exposure of granulosa cells to PA inhibits cell proliferation and decreases steroidogenesis. PA impairs fertility by suppressing human granulosa cell survival and inducing apoptosis [13, 14]. Therefore, ameliorating the toxic effects of PA on granulosa cells may be an effective method to treat human infertility. To date, the exact molecular mechanism of PA-induced granulosa cell apoptosis, however, has not been fully understood. Our previous studies have suggested that ER stress is involved in granulosa cell apoptosis [15, 16]. However, it remains elusive whether ER stress is involved in PA-induced granulosa cell apoptosis.

The ER plays an important role in the folding, transport, and processing of newly synthesized proteins and the biosynthesis of cholesterol, steroids, and other lipids, which is essential for the maintenance of homeostasis in organisms. The accumulation of unfolded or misfolded proteins in the ER lumen can affect ER homeostasis and trigger a protective mechanism known as the unfolded protein response (UPR). Three ER transmembrane proteins, protein kinase RNA (PKR)-like ER kinase (PERK), inositol-requiring enzyme-1 (IRE-1), and activating transcription factor-6 (ATF6), are involved in ER stress and are associated with glucose-regulated protein 78 (GRP78, an ER chaperone) [17]. The primary objective of the UPR is to re-establish homeostasis and alleviate ER stress by increasing the protein folding capacity and decreasing the unfolded protein load. However, when ER stress fails to manage misfolded and unfolded proteins, cell apoptosis is induced [18]. Previous studies have reported that melatonin inhibits cell apoptosis by attenuating ER stress [19–21].

Melatonin is an important endogenous hormone involved in the biological clock, the circadian rhythm and reproductive physiology. Its actions are mediated via two types of receptors, MT1 and MT2, which are expressed in not only the pineal gland but also other parts of the organism, including granulosa cells [22–24]. Increasing evidence from in vitro cultured cell and animal studies has shown the beneficial effects of melatonin on female reproductive processes, such as follicle growth [25, 26], embryonic development [27] and oocyte maturation [25]. Dynamic changes in the porcine intrafollicular melatonin concentration correlate with the progress of follicular atresia. Normally, melatonin levels might positively correlate with follicular growth [28]. High levels of melatonin were found in human preovulatory FF [29]. A recent study revealed that the intrafollicular melatonin concentration decreases as follicular atresia progresses, whereas the percentage of apoptotic granulosa cells increases [26]. The initiation of granulosa cell apoptosis during porcine follicular atresia may be related to an ER stress response, and melatonin can inhibit apoptosis and stimulate progesterone production by granulosa cells [26, 30]. PA has been demonstrated to induce apoptosis in human granulosa cells [13]. However, the effects and mechanisms of melatonin on PA-induced granulosa cell apoptosis have never been studied.

In this study, we aimed to explore the potential molecular mechanisms of PA-induced apoptosis, to detect the effects of melatonin on PA-induced apoptosis, and to examine whether this process is related to ER stress in mouse granulosa cells.

## Materials and methods

### Chemicals and mice

All chemicals were purchased from Sigma-Aldrich (St. Louis, MO, USA) unless otherwise stated. Female Kunming White outbred strain mice (21-28 days old) were obtained from the Experimental Animal Center of Jiujiang University. All mice were fed a typical diet of lab chow and housed in a single room under conditions of constant temperature (25-28 ± 2 °C), humidity (55 ± 5%), and lighting (12 h light, 12 h dark cycle). All procedures were approved by the Committee for the Ethics on Animal Care and Experiments of Jiujiang University (approval No. SYXK(GAN)2017-0001).

### Granulosa cell collection, culture and treatment

Immature female mice (*n* = 285) were intraperitoneally injected with 8 IU pregnant mare serum gonadotrophin (PMSG) (Ningbo Sansheng, Ningbo, China) to facilitate granulosa cell proliferation. After 44 h, primary mouse granulosa cells were isolated and cultured as described previously [31]. Briefly, the ovaries were collected, and granulosa cells were released by puncturing the follicles with 26-gauge needles under sterile conditions. Then, the granulosa cells were washed and collected by brief centrifugation, and cell viability was determined by trypan blue exclusion. Finally, the cells were cultured in Dulbecco’s modified Eagle’s medium/Ham’s F 12 nutrient mixture (DMEM/F12, HyClone, UT, USA) containing 100 IU/mL penicillin, 100 μg/mL streptomycin, and 10% fetal bovine serum (FBS, Corning, USA) at 37 °C in 5% CO_2_ and 95% O_2_ for 48 h.

**Fig. 1:**
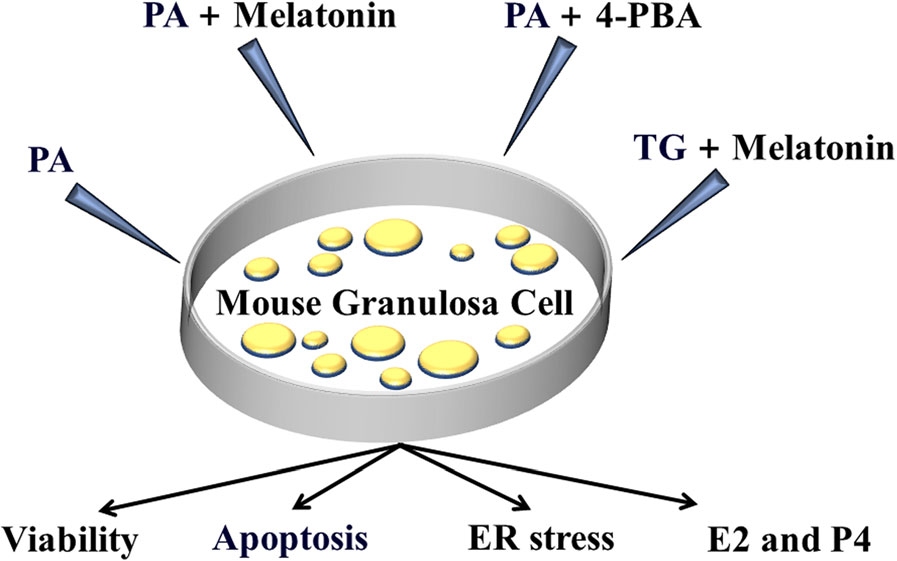
Schematic of the experimental design. Mouse granulosa cells were collected from the ovaries of immature female mice and then treated with PA, PA + melatonin, PA + 4-PBA or TG + melatonin to explore the mechanism of melatonin that protects against PA-induced cell apoptosis

Melatonin was freshly dissolved in absolute ethanol to produce a 100 mM stock solution and then kept at 4 °C. Before application, stock solution was further diluted in culture medium. 4-Phenylbutyrate (4-PBA, ER stress inhibitor), and thapsigargin (TG, ER stress agonist) were prepared in dimethyl sulfoxide (DMSO) and immediately diluted with culture medium before the experiment. The final concentration of DMSO in the incubation mixture did not exceed 0.1%. When the cells reached 70-80% confluence, they were exposed to increasing concentrations of melatonin (0.1-100 μM) or PA (100-800 μM). In another experiment, cells were exposed to TG (500 nM) and PA (400 μM) in the absence or presence of 4-PBA (500 nM) and melatonin (10 μM) (Fig. 1). Standard conditions were maintained for the control group. After incubation for 24 h, the cells were collected to assess cell viability, apoptosis, and Caspase 3 activity, and western blotting was performed.

### Cell proliferation assay

Granulosa cells (*n* = 35 mice) were plated into 96-well plates at 1 × 10^5^ cells/mL. After treatment, the cells were treated with 0.5 mg/mL MTT and then incubated for an additional 4 h. At the end of the experiments, the cell growth medium was replaced with 150 μL DMSO and colorimetric measurements were performed with an ELISA plate reader at 570 nm [32]. The experiments were performed in triplicate (about 12 mice in a group). Cell viability was calculated as follows: relative viability (%) = [A450 (treated) - A450 (blank)] / [A450 (control) - A450 (blank)] × 100%.

### Measurement of the estradiol and progesterone levels

Briefly, granulosa cells (*n* = 45 mice, about 15 mice in each group) were cultured in DMEM/F12 for 48 h. The cells were then incubated with PA, TG, 4-PBA or/and melatonin for 48 h. After 48 h of treatment, the mouse granulosa cells were counted. Then, the concentrations of E2 and P4 in the culture supernatants were measured with ELISA kits (Ji Yin Mei, Wuhan, China) according to the manufacturer’s instructions. The minimum detectable concentration of E2 was 10 pg/mL. The intra- and interassay coefficients of variation were < 10 and < 15%, respectively. The minimum detectable concentration of P4 was 0.25 ng/mL. The intra- and interassay coefficients of variation were < 10 and < 15%, respectively. Each sample was measured in triplicate.

### Cell apoptosis measurement

The apoptosis level of cells (*n* = 55 mice, about 18 mice in each group) was quantified with an Annexin V/PI Apoptosis Analysis Kit according to the manufacturer’s instructions. The cells were analyzed using a fluorescence-activated cell sorter (Becton, Dickinson and Company, USA) within 1 h of staining. The proportion of early apoptotic cells was determined by measuring the percentage of Annexin V+/PI− cells; the proportion of progressed apoptotic cells was obtained by determining the percentage of Annexin V+/PI+ cells; the proportion of necrotic cells was detected by determining the percentage of Annexin V−/PI+ cells and Annexin V−/PI− cells were considered surviving cells as previously reported [18].

### Western blot analysis

After treatment, cells (*n* = 150 mice, about 50 mice in each group) were collected and washed with an ice-cold phosphate buffer solution (PBS) and lysed with radioimmunoprecipitation assay (RIPA) lysis buffer containing 1% phenylmethylsulfonyl fluoride (PMSF), and the total protein concentration was measured by a bicinchoninic acid (BCA) assay according to the manufacturer’s instructions. For each sample, 30 μg total protein was separated on a 12% polyacrylamide gel before being transferred to polyvinylidene difluoride (PVDF) membranes (Millipore, Bedford, MA, USA). After blocking in Tris-buffered saline-Tween-20 (TBST) supplemented with 5% skim milk at 25 °C for 1 h, the membranes were incubated overnight at 4 °C with a primary antibody (Additional file 1: Table S1). After washing, the membranes were incubated with a secondary antibody conjugated with horseradish peroxidase at 37 °C for 30 min. Finally, the immunoreactive bands were visualized using a Super Signal West Pico kit (Proteintech, Wuhan, China) with a Bio-Rad imaging system (Bio-Rad, CA, USA) according to the manufacturer’s instructions. The protein band densities were semiquantified by densitometric analysis using ImageJ software 1.48 (Bethesda, MD, USA).

### Statistical analyses

All experiments were repeated three times for each group, and the data are presented as the mean ± SEM. The data were analyzed by ANOVA, followed by Fisher’s least significant difference test and independent samples Student’s t test, with SPSS software, version 13.0 (SPSS, Chicago, IL, USA).

## Results

### Effects of PA on cell viability and apoptosis in mouse granulosa cells

**Fig. 2:**
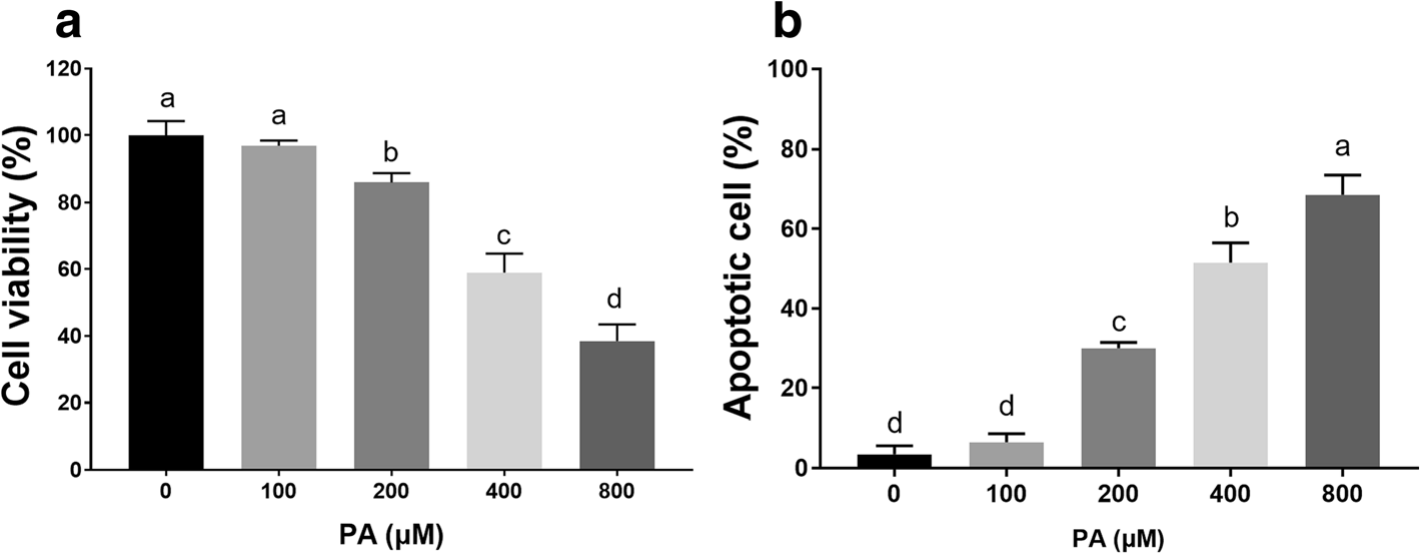
Effect of PA on the growth and apoptosis of mouse granulosa cells. **a** Cells were treated with different concentrations (100-800 μM) of PA for 24 h and then processed for the cell activity analysis. **b** Cells were treated with different concentrations (100-800 μM) of PA for 24 h and then processed for an apoptosis assay. Data are presented as the mean ± SEM of three independent experiments. Bars with different letters are significantly different (*p* < 0.05)

The effect of PA on mouse granulosa cells was examined by treatment with 100-800 μM PA for 24 h. The MTT results showed that PA had very little effect at the low doses of 100 μM, while 200-800 μM PA significantly inhibited cell viability (Fig. 2a). We further analyzed the granulosa cell apoptosis induced by PA via flow cytometry. The results revealed that compared with the control treatment, PA treatment increased the percentage of apoptotic granulosa cells in a dose-dependent manner (Fig. 2b), and the IC50 value was approximately 400 μM.

### Effect of PA on caspase 3 activity and BAX expression in mouse granulosa cells

**Fig. 3:**
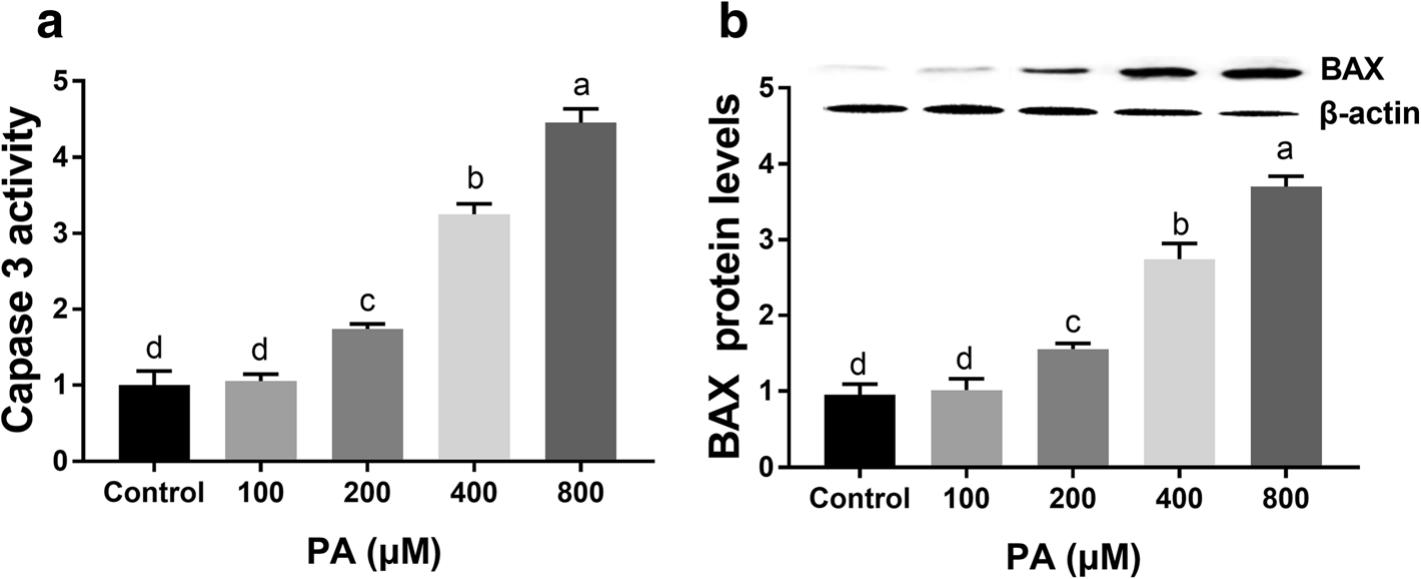
Effect of PA on apoptosis-related gene expression in mouse granulosa cells. **a** Caspase 3 activity in mouse granulosa cells after treatment with different doses of PA (0-800 μM) for 24 h. **b** Relative BAX expression in mouse granulosa cells after treatment with different doses of PA (0-800 μM) for 24 h. BAX expression was normalized to the level of β-actin expression. The statistical analysis results are shown in the bar graphs. Data are presented as the mean ± SEM of three independent experiments. Bars with different letters are significantly different (*p* < 0.05)

To further explore the mechanism of PA-induced cell apoptosis in granulosa cells, the expression of apoptosis-related genes (Caspase 3 and BAX) was measured by a colorimetric assay and western blot analysis after treatment with 100-800 μM PA for 24 h. The results suggested that the expression patterns of Caspase 3 and BAX were similar, and the expression of both molecules increased in a dose-dependent manner after treatment for 24 h. The minimum effective dose was 200 μM PA (Fig. 3).

### Effect of PA on GRP78 and CHOP expression in mouse granulosa cells

**Fig. 4:**
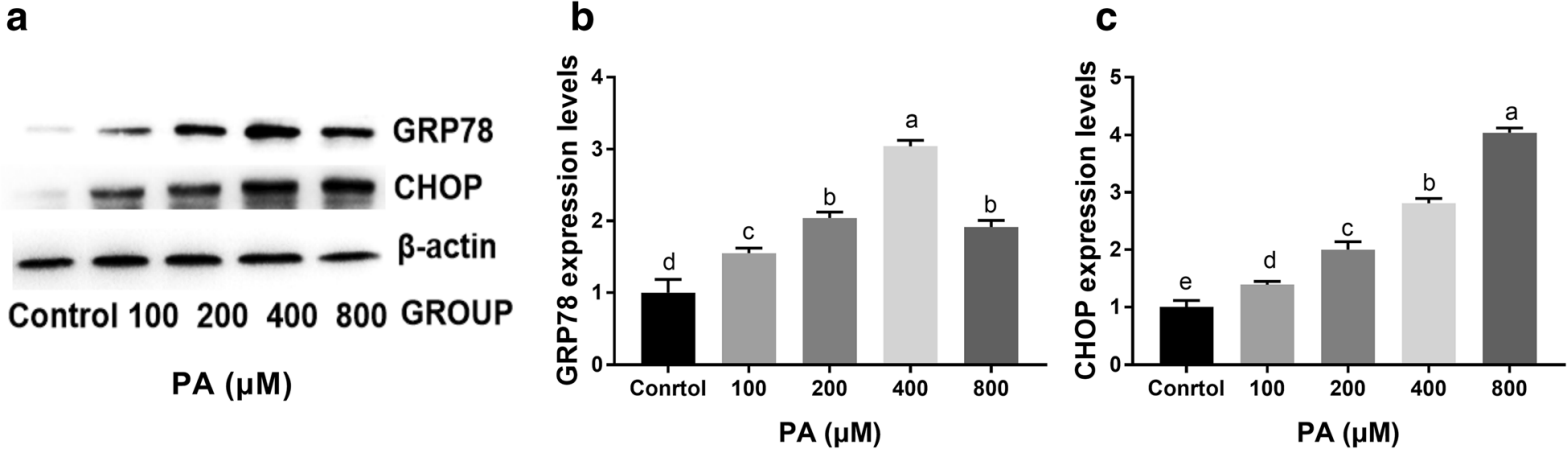
Effect of PA on ER stress-related protein expression in mouse granulosa cells. **a** Expression of GRP78 and CHOP in mouse granulosa cells after treatment with different doses of PA (100-800 μM) for 24 h. **b** Relative expression of GRP78 after treatment with different doses of PA (100-800 μM) for 24 h. **c** Relative expression of CHOP after treatment with different doses of PA (100-800 μM) for 24 h. Protein expression was normalized to the level of β-actin expression. The statistical analysis results are shown in the bar graphs. Data are presented as the mean ± SEM of three independent experiments. Bars with different letters are significantly different (*p* < 0.05)

We detected the expression of ER stress marker genes (GRP78 and CHOP) after treatment with 100-800 μM PA for 24 h. The results showed that PA treatment at a concentration up to 400 μM significantly induced the expression of GRP78 in a dose-dependent manner. However, 800 μM PA decreased GRP78 expression at 24 h (Fig. 4a and b). CHOP expression was significantly induced by 200 μM PA, and 800 μM PA induced the highest expression levels at 24 h (Fig. 4a and c).

### Melatonin attenuated the cytotoxicity of PA in mouse granulosa cells

**Fig. 5:**
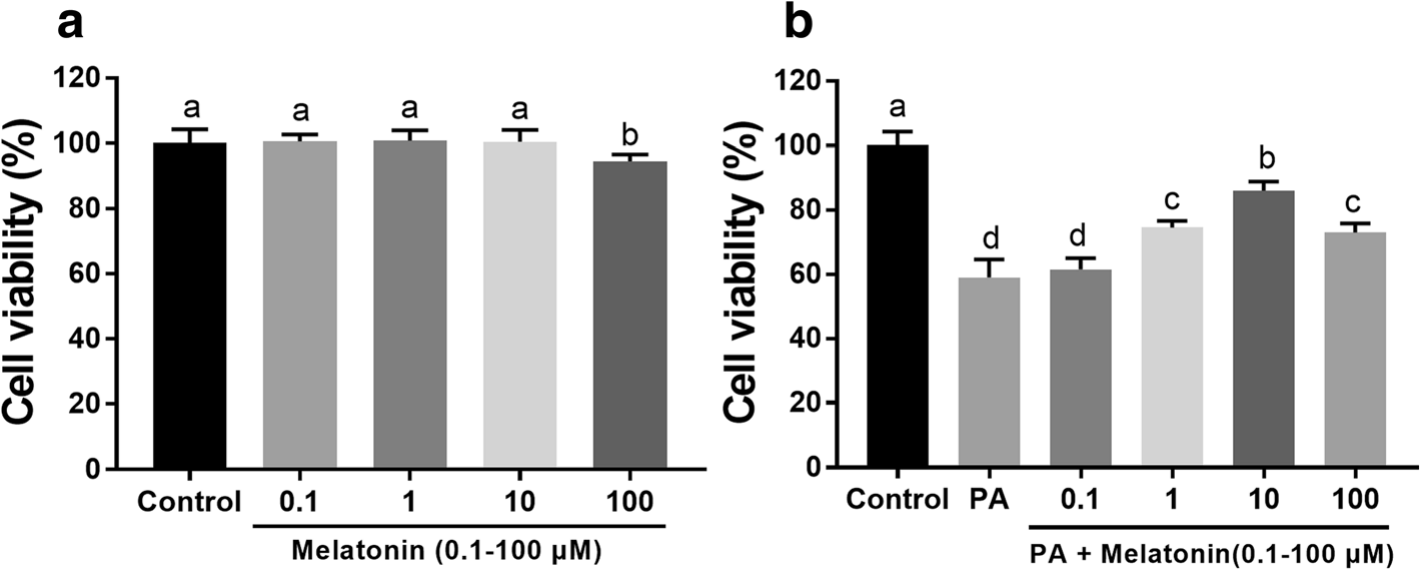
Effects of melatonin on PA-induced mouse granulosa cell activity. **a** Cells were treated with different concentrations of melatonin (0.1-100 μM) for 24 h and then processed for cell activity analysis. **b** Cells were treated with different concentrations of melatonin (0.1-100 μM) and 400 μM PA for 24 h. Data are presented as the mean ± SEM of three independent experiments. Bars with different letters are significantly different (*p* < 0.05)

We first detected the 0.1-100 μM melatonin on the viability of mouse granulosa cells at 24 h. The results showed that melatonin alone had no effect on the viability of granulosa cells at concentrations up to 10 μM, whereas the 100 μM melatonin group showed decreased cell viability compared to the control group (Fig. 5a). Compared with the 400 μM PA group, the 1 and 10 μM melatonin groups exhibited a dose-dependent enhancement in cell viability; however, the 100 μM melatonin group had lower cell viability than the 10 μM group and higher viability than the PA-treated group (Fig. 5b).

### Melatonin inhibited PA-induced apoptosis in mouse granulosa cells

**Fig. 6:**
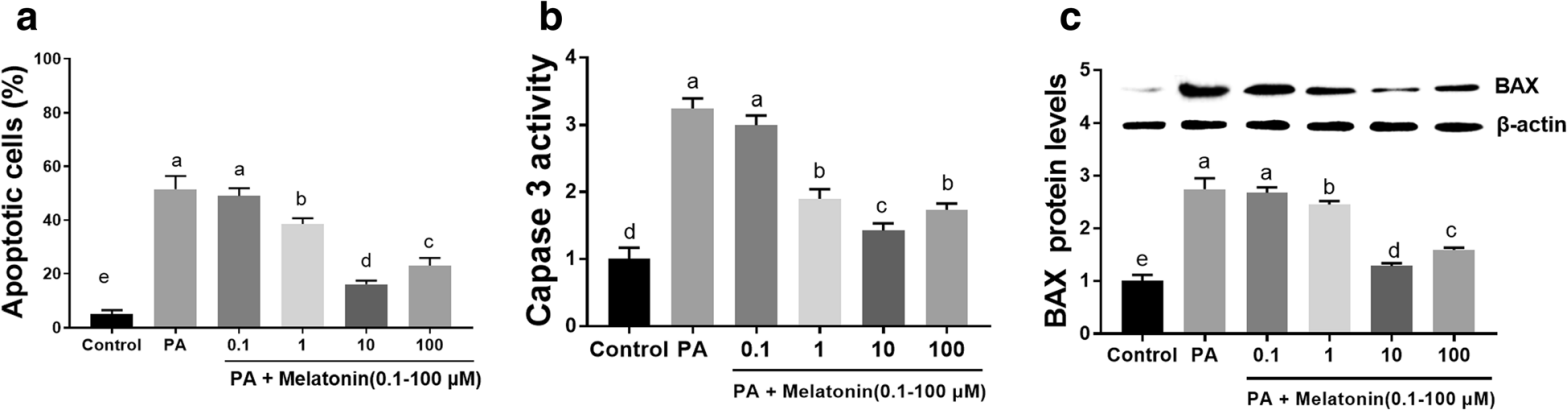
Effects of melatonin on PA-induced mouse granulosa cell apoptosis and apoptosis-related gene expression. **a** Effects of different concentrations of melatonin (0.1-100 μM) on PA-induced mouse granulosa cell apoptosis after 24 h. **b** Caspase 3 activity in mouse granulosa cells after treatment with different concentrations of melatonin (0.1-100 μM) and 400 μM PA for 24 h. **c** Relative BAX expression in mouse granulosa cells after treatment with different concentrations of melatonin (0.1-100 μM) and 400 μM PA for 24 h. BAX expression was normalized to the level of β-actin expression. The statistical analysis results are shown in the bar graphs. Data are presented as the mean ± SEM of three independent experiments. Bars with different letters are significantly different (*p* < 0.05)

To detect the cytoprotective effect of melatonin on PA-treated granulosa cells, cells were treated with 400 μM PA and 0.1-100 μM melatonin for 24 h. The flow cytometry results showed that melatonin treatment obviously reduced the cellular apoptosis rate, and the lowest effective concentration was 1 μM (Fig. 6a). We also detected Caspase 3 and BAX expression by a colorimetric assay and western blot analysis. Similar to the flow cytometry results, these results revealed that melatonin treatment successfully reduced the Caspase 3 activity and BAX protein expression caused by 400 μM PA (Fig. 6b and c).

### Melatonin suppressed PA-induced ER stress in mouse granulosa cells

**Fig. 7:**
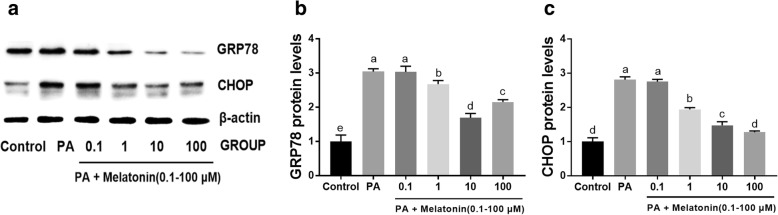
Effects of melatonin on the expression of the ER stress marker genes GRP78 and CHOP during PA-induced mouse granulosa cell apoptosis after treatment for 24 h. **a** Western blot assays of GRP78 and CHOP expression after treatment with different concentrations of melatonin (0.1-100 μM) and 400 μM PA for 24 h. **b** Relative GRP78 expression in mouse granulosa cells after treatment with different concentrations of melatonin (0.1-100 μM) and 400 μM PA for 24 h. **c** Relative CHOP expression in H9C2 cells after treatment with different concentrations of melatonin (0.1-100 μM) and 400 μM PA for 24 h. GRP78 and CHOP expression were normalized to the level of β-actin expression. The Statistical analysis results are shown in the bar graphs. Data are presented as the mean ± SEM of three independent experiments. Bars with different letters are significantly different (*p* < 0.05)

To detect the effect of melatonin on PA-induced ER stress, mouse granulosa cells were treated with 400 μM PA and 0.1-100 μM melatonin for 24 h. Then, CHOP and GRP78 expression was detected by western blot analysis. The results showed that PA markedly enhanced the expression of GRP78 and CHOP, and these expression levels were attenuated by melatonin in a concentration-dependent manner up to a concentration of 10 μM (Fig. 7).

### 4-PBA attenuated PA-induced cytotoxicity, apoptosis and ER stress in mouse granulosa cells

**Fig. 8:**
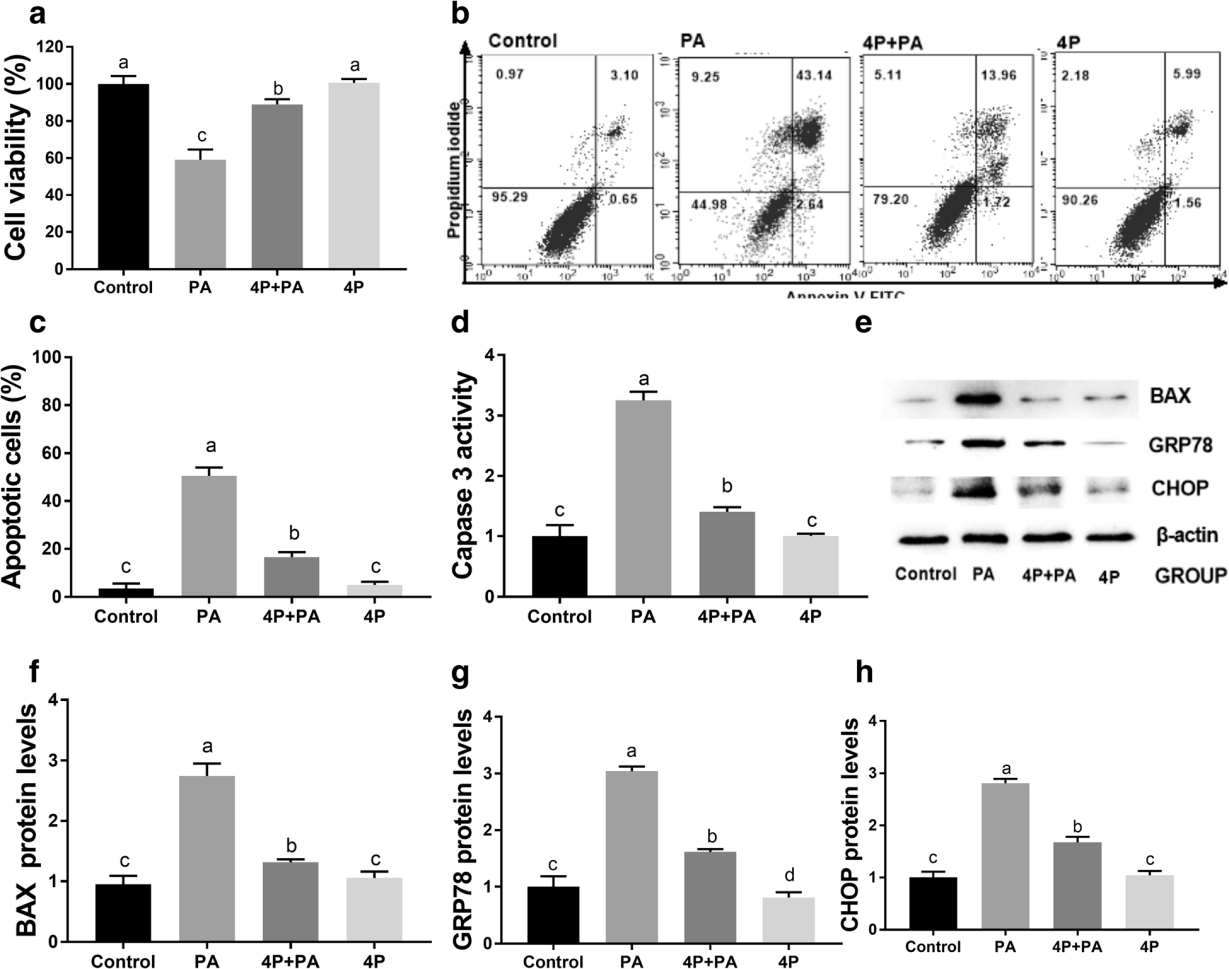
Effects of 4-PBA on PA-treated mouse granulosa cell viability, cell apoptosis and ER stress at 24 h. **a** Cell viability was measured by an MTT assay. **b**, **c** Apoptosis was detected via flow cytometry. **d** Western blot analysis was used to measure BAX, GRP78 and CHOP expression. **e** The Caspase 3 activity and (**f**) the relative BAX expression of mouse granulosa cells were measured. **g** The relative GRP78 and (**h**) CHOP expression was measured. The protein expression levels were normalized to the level of β-actin. PA: 400 μM palmitic acid; 4P: 500 nM 4-PBA. The statistical analysis results are shown in the bar graphs. Data are presented as the mean ± SEM of three independent experiments. Bars with different letters are significantly different (*p* < 0.05)

To confirm the role of ER stress in PA-mediated mouse granulosa cell apoptosis, cells were treated with PA and the ER stress inhibitor 4-PBA. The results revealed that 4-PBA significantly promoted cell viability and prevented the cell apoptosis caused by PA at 24 h (Fig. 8a-c). In addition, 4-PBA obviously reduced Caspase 3 activity and BAX expression (Fig. 8d-f). In addition, western blotting revealed that the expression levels of GRP78 and CHOP in the PA-treated cells were markedly decreased after treatment with 4-PBA (Fig. 8e-h).

### Melatonin rescued TG-induced decreased cell viability, apoptosis, and ER stress in mouse granulosa cells

**Fig. 9:**
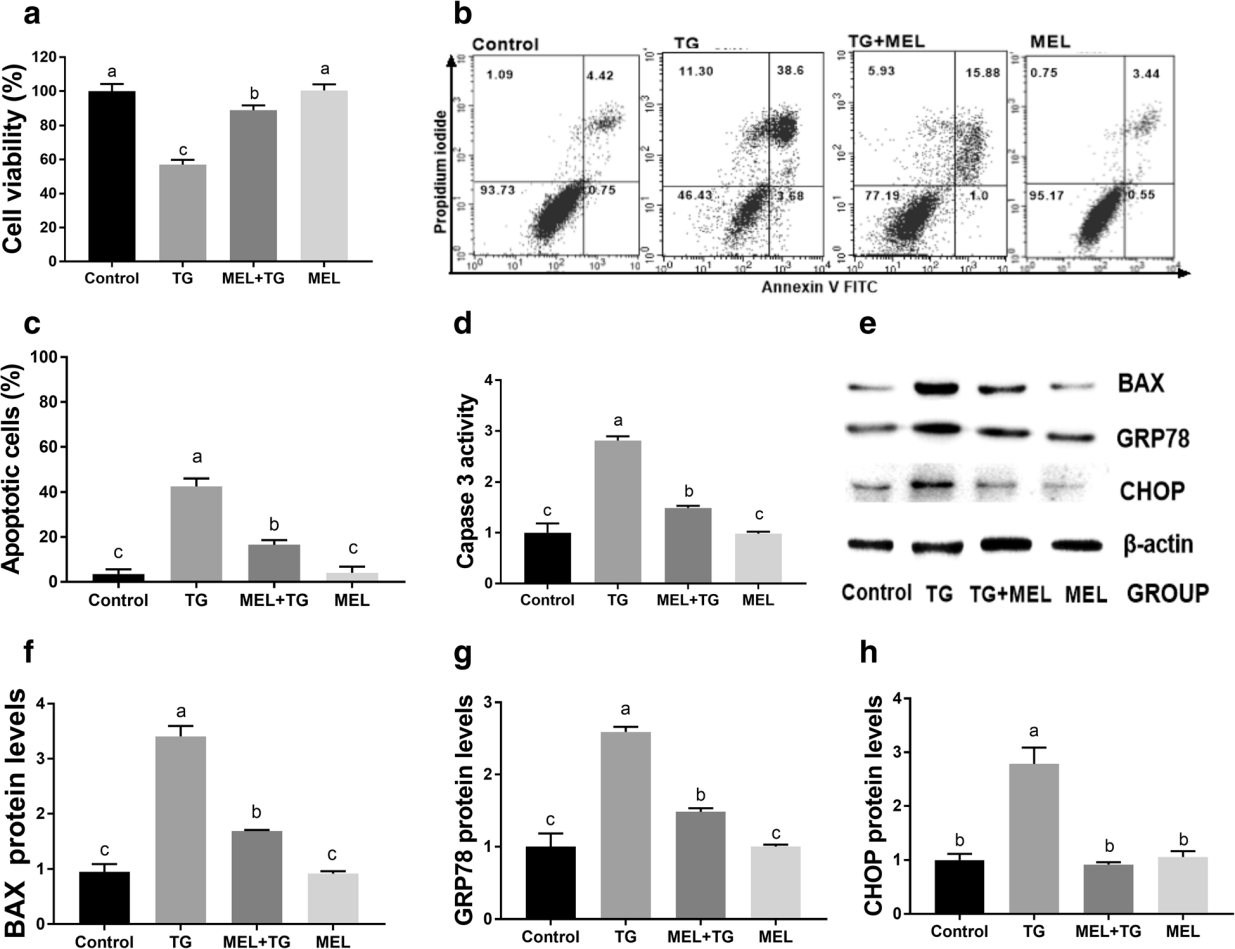
Effects of melatonin on TG-treated mouse granulosa cell viability, cell apoptosis and ER stress at 24 h. **a** Cell viability measured by an MTT assay. **b**, **c** Apoptosis detected via flow cytometry. **d** Western blot assays of BAX, GRP78 and CHOP expression. **e** Caspase 3 activity of H9C2 cells. **f** Relative BAX expression. **g** Relative GRP78 expression. **h** Relative CHOP expression. The protein expression levels were normalized to the β-actin level. PA: 400 μM palmitic acid; TG: 500 nM thapsigargin; MEL: 10 μM melatonin; 4P: 500 nM 4-PBA. The statistical analysis results are shown in the bar graphs. Data are presented as the mean ± SEM of three independent experiments. Bars with different letters are significantly different (*p* < 0.05)

To detect the effect of melatonin on ER stress-induced cell apoptosis, we added 500 nM TG to the culture medium to induce ER stress. Treatment with 10 μM melatonin significantly promoted cell viability and prevented the cell apoptosis induced by TG (Fig. 9a-c). The western blotting results indicated that melatonin reduced the protein levels of GRP78 and CHOP in the TG-treated mouse granulosa cells (Fig. 9e, g and h). We also detected the expression of Caspase 3 and BAX. The results showed that PA significantly increased the protein expression levels of Caspase 3 and BAX compared with the control treatment (Fig. 9d-f).

### Melatonin attenuated the decreases in the E2 and P4 levels in PA-treated mouse granulosa cells

**Fig. 10:**
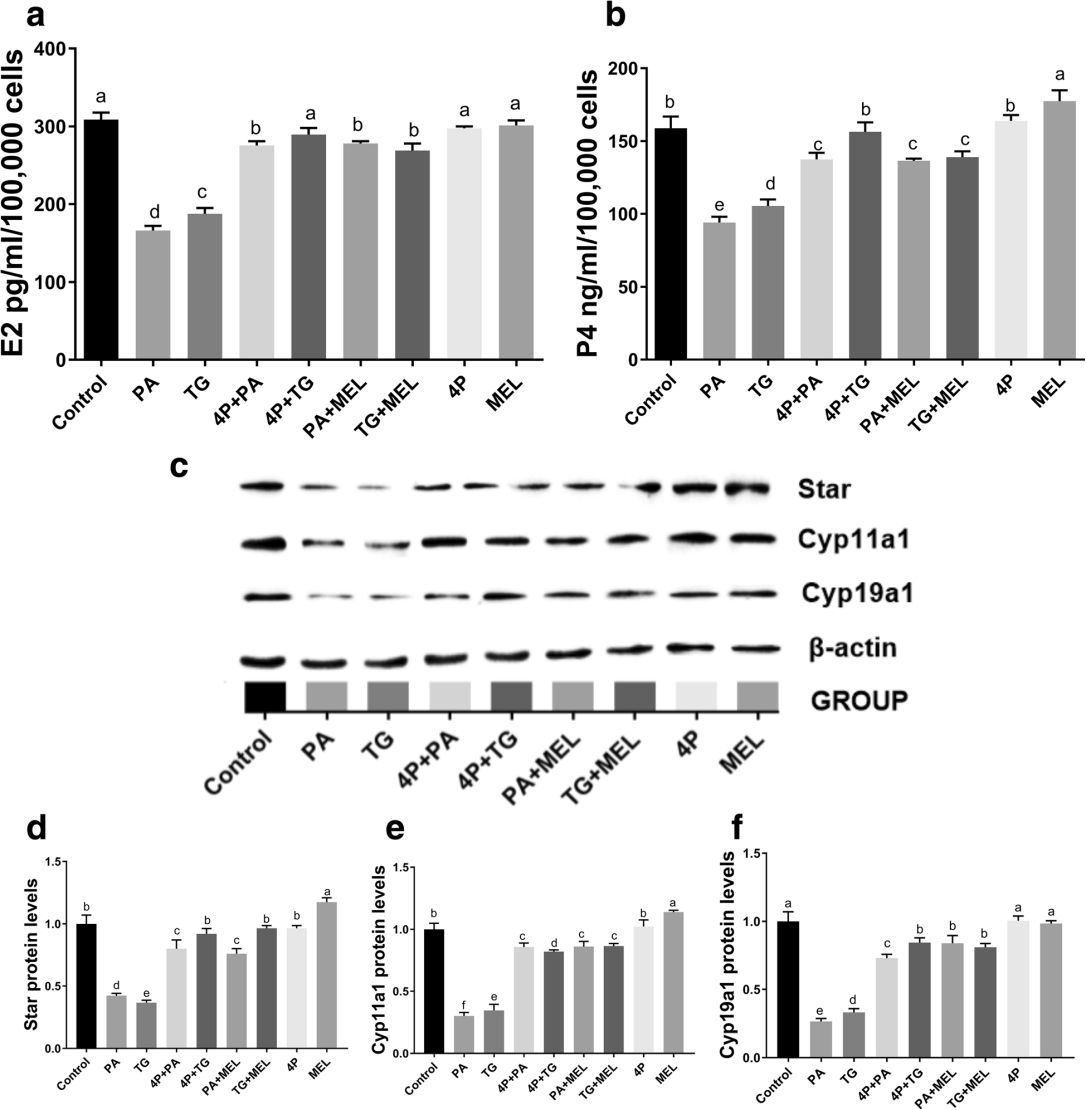
Effects of melatonin on E2 and P4 generation in PA- or TG-treated mouse granulosa cells. **a** E2 levels in mouse granulosa cell culture medium after 48 h of culture. **b** P4 levels in mouse granulosa cell culture medium after 48 h of culture. **c** Western blot assays for Star, Cyp11a1 and Cyp19a1 expression at 24 h. **d** Relative Star expression. **e** Relative Cyp11a1 expression. **f** Relative Cyp19a1 expression. The protein expression levels were normalized to the β-actin level. CTL: control; TG: 500 nM thapsigargin; MEL: 10 μM melatonin. The statistical analysis results are shown in the bar graphs. Data are presented as the mean ± SEM of three independent experiments. Bars with different letters are significantly different (*p* < 0.05)

To assess the effects of melatonin on steroid hormones in PA-induced mouse granulosa cells, we measured the concentrations of E2 and P4 in the culture medium after PA and TG treatment. The results showed that 500 nM TG and 400 μM PA inhibited the expression of steroidogenic enzymes expression (Star, Cyp19a1 and Cyp11a1) and the production of steroid hormones (E2 and P4) (Fig. 10). In contrast, 10 μM melatonin treatment slightly increased the expression of Star and Cyp11a1 but not Cyp19a1 and enhanced the synthesis of P4 (Fig. 10a-f). Additionally, melatonin increased the levels of E2 and P4 generation and the expression of steroidogenic enzymes including Star, Cyp19a1 and Cyp11a1 induced by PA or TG (Fig. 10a-f).

## Discussion

PA is a common saturated fatty acid and the PA level in mammalian FF is reported to be approximately 10^− 4^ M. A previous study showed that PA induces granulosa cell apoptosis and inhibits steroidogenesis [13]. Our previous studies found that ER stress is related to granulosa cell apoptosis and steroidogenesis [15, 16]. In addition, some studies have reported that melatonin inhibits cell apoptosis through attenuating ER stress [19–21]. In this study, we showed that PA-induced granulosa cell apoptosis and steroidogenesis downregulation occur via ER stress and that melatonin can rescue this process.

We first detected the effects of different concentrations of PA on cell viability and apoptosis. The data from our study showed that cell viability was significantly decreased and apoptosis was induced during in vitro culture with PA. Our study was consistent with a previous study showing that PA markedly inhibits the proliferation of human granulosa cells in a dose-dependent manner [13]. We also measured the expression of apoptosis-related genes (Caspase 3 and BAX) during PA treatment. Caspase 3 acts as an executioner in caspase-mediated apoptosis, and the expression of Caspase 3 positively correlates with the rate of apoptosis in cells [33]. Moreover, BAX, a member of the BCL2 family, has a proapoptotic effect [34]. In the present study, we found that Caspase 3 activation and BAX expression were upregulated after PA treatment. Based on these results, we concluded that PA can regulate Caspase 3 activation and BAX expression to affect cell apoptosis in mouse granulosa cells.

Furthermore, we detected GRP78 and CHOP expression when cells were cultured with 100-800 μM PA for 24 h. The results showed that PA treatment induced the expression of GRP78 and CHOP, which indicated that PA activates the ER stress pathway in cultured mouse granulosa cells. GRP78 is an ER stress-response protein, and its expression is a typical marker of ER stress activation. Our previous results showed that PA triggers ER stress and then induces apoptosis though CHOP activation in Saos-2 cells [18]. In addition, this result is consistent with the results of other studies that showed that PA induces ER stress and apoptosis in hepatoma cells [35] and myocardial cells [34]. The present study further demonstrated PA-induced ER stress in mouse granulosa cells. To our knowledge, this is the first study to demonstrate that ER stress can be induced by PA in granulosa cells, which implies that ER stress is involved in PA-induced granulosa cell apoptosis.

Melatonin is an important endogenous hormone and exists in mammalian FF. Physiologically, the concentration of melatonin in mammalian follicular fluid is reported to be approximately 10^− 11^ M. However, the melatonin level in the FF is reported to be dynamic and reaches its zenith in the preovulatory follicle [36–40]. Numerous studies have revealed that melatonin exerts various biological effects, such as antioxidative [41], anti-inflammatory [42] and anti-apoptotic effects [43]. Furthermore, ER stress plays a key role in the process of cell injury, and some studies also reported that melatonin inhibits cell apoptosis via the ER stress pathway [19–21]. Interestingly, there is evidence suggesting that that intrafollicular melatonin concentration decreased as follicular atresia progressed whereas the percentage of apoptotic granulosa cells increased [26]. Thus, we suspected that dynamic change of melatonin and PA level in FF may be closely related to granulosa cell apoptosis. Therefore, we identified the effects of melatonin on granulosa cell apoptosis and ER stress. Our results suggested that melatonin obviously inhibited PA- or TG-induced GRP78 and CHOP expression. Furthermore, melatonin remarkably inhibited PA- or TG-induced mouse granulosa cell apoptosis and the expression of Caspase 3 and BAX, suggesting that melatonin can protect cells against PA and ER stress-induced cell apoptosis. To further confirm the role of ER stress in PA-induced granulosa cell apoptosis, we added the ER stress inhibitor 4-PBA to culture medium containing PA. The results showed that 4-PBA and melatonin both suppressed PA-induced cell apoptosis and decreased the expression of GRP78 and CHOP. This date further confirmed the fact that melatonin significantly reduced the cell apoptosis induced by PA via the ER stress pathway.

Granulosa cells play an important role in hormonal synthesis in females. Compared with the control treatment, PA treatment decreased E2 and P4 expression. The possible reason for the reductions could be the decreases in the levels of Star (the protein associated with the transport of cholesterol across the mitochondrial membrane), Cyp11a1 (the monooxygenase that catalyzes cholesterol and steroid synthesis), and Cyp19a1 (the enzyme responsible for androgen aromatization to estrogen) [31] shown in our results. Previous studies have demonstrated that E2 and P4 are indispensable for maintaining the normal physiological function of the ovary [44]. Thus, we infer that PA may induce follicular atresia and affect ovulation by regulating P4 and E2 production in granulosa cells.

In agreement with its effect on PA-induced cell apoptosis, melatonin and 4-PBA also reverse the PA-mediated inhibition of hormone secretion. We also found that the PA-induced decrease in hormone secretion-related protein expression was partly inhibited by melatonin or 4-PBA, indicating that melatonin improves hormone secretion in PA- or TG-treated mouse granulosa cells. This result further confirmed the findings of previous studies that showed that melatonin weakens ER stress [19–21] and ER stress is involved in granulosa cell apoptosis [15, 16] and hormone secretion [44].

The concentration of melatonin used in the present study is 10^− 6^ M which seems to be higher than physical level. However, we thought that the high concentration of melatonin used in this study could reflect the in vivo conditions. As we known, melatonin is considered as a broad-spectrum antioxidant. After melatonin reacts with ROS, the melatonin metabolites are produced and accumulated in vivo. Subsequently, the melatonin metabolites also work as antioxidants, resulting in melatonin and melatonin metabolites working together as powerful antioxidants in vivo [43]. Accumulating evidence indicates that PA increases the production of reactive oxygen species (ROS) in granulosa cells and that oxidative stress is essential for the induction of ER stress in granulosa cells [45]. Therefore, we speculated that PA might induce oxidative stress by stimulating the generation of ROS, destroying the antioxidant capacity, and subsequently triggering ER stress and apoptosis in granulosa cells. Melatonin and its metabolites may effectively decrease the PA-induced generation of ROS to mitigate oxidative stress, protecting granulosa cells from PA-induced apoptosis through ER stress.

## Conclusion

In summary, our results initially demonstrated that PA-induced cell mouse granulosa cell apoptosis occurs via the ER stress pathway. Furthermore, melatonin has the potential to protect mouse granulosa cells from PA-induced cell apoptosis and hormone expression downregulation. Therefore, melatonin is a potent agent that could be exploited as a potential intervention in the prevention of some reproductive abnormalities, especially in female infertility induced by saturated fatty acids.

## Additional file

Additional file 1: **Table S1.** Antibody details. (DOCX 16 kb)

## Abbreviations

ATF6: activating transcription factor-6
BAX: B-cell lymphoma-2 (BCL-2) associated X protein
BCA: bicinchoninic acid
CHOP: CCAAT/enhancer binding protein homologous protein
DMSO: dimethyl sulfoxide
E2: estrogen
ER: endoplasmic reticulum
FF: follicular fluid
GRP78: glucose-regulated protein 78
IRE-1: inositol-requiring enzyme-1
MTT: methyl thiazolyl tetrazolium
P4: progesterone
PA: palmitic acid
PBS: phosphate buffer solution
PERK: protein kinase RNA (PKR)-like ER kinase
PMSG: pregnant mare serum gonadotrophin
PVDF: polyvinylidene difluoride
RIPA: radioimmunoprecipitation assay
ROS: reactive oxygen species
TBST: Tris-buffered saline-Tween-20
TG: thapsigargin
UPR: unfolded protein response
