# Supplementary material for: Melatonin attenuates palmitic acid-induced mouse granulosa cells apoptosis via endoplasmic reticulum stress
Source: J Ovarian Res. 2019 May 10;12:43. doi: 10.1186/s13048-019-0519-z (PMC6511168; doi:10.1186/s13048-019-0519-z)
Supplement: Supplementary file 1 — Table S1. Antibody details. (DOCX 16 kb) [file 13048_2019_519_MOESM1_ESM.docx]

Additional file 1: **Table S1.** Antibody details.

| **Target(diluted)** | **Catalogue number** | **Company** |
| --- | --- | --- |
| CHOP(1:1000) | ab10444 | Abcam |
| GRP78(1:1000) | ab32618 | Abcam |
| BAX(1:500) | sc-4239 | Santa Cruz |
| Star(1:500) | sc-166821 | Santa Cruz |
| Cyp11a1(1:500) | sc-18043 | Santa Cruz |
| Cyp19a1(1:500) | sc-14244 | Santa Cruz |
| β-actin(1:2000) | sc-47724 | Santa Cruz |
